# Peptide-Major Histocompatibility Complex Class I Binding Prediction Based on Deep Learning With Novel Feature

**DOI:** 10.3389/fgene.2019.01191

**Published:** 2019-11-28

**Authors:** Tianyi Zhao, Liang Cheng, Tianyi Zang, Yang Hu

**Affiliations:** ^1^Department of Computer Science and Technology, School of Life Science and Technology, Harbin Institute of Technology, Harbin, China; ^2^College of Bioinformatics Science and Technology, Harbin Medical University, Harbin, China

**Keywords:** peptide-major histocompatibility complex class I binding prediction, deep learning, convolutional neural network, epitope prediction, human leukocyte antigen

## Abstract

Peptide-based vaccine development needs accurate prediction of the binding affinity between major histocompatibility complex I (MHC I) proteins and their peptide ligands. Nowadays more and more machine learning methods have been developed to predict binding affinity and some of them have become the popular tools. However most of them are designed by the shallow neural networks. Bengio said that deep neural networks can learn better fits with less data than shallow neural networks. In our case, some of the alleles only have dozens of peptide data. In addition, we transform each peptide into a characteristic matrix and input it into the model. As we know when dealing with the problem that the input is a matrix, convolutional neural network (CNN) can find the most critical features by itself. Obviously, compared with the traditional neural network model, CNN is more suitable for predicting binding affinity. Different from the previous studies which are based on blocks substitution matrix (BLOSUM), we used novel feature to do the prediction. Since we consider that the order of the sequence, hydropathy index, polarity and the length of the peptide could affect the binding affinity and the properties of these amino acids are key factors for their binding to MHC, we extracted these information from each peptide. In order to make full use of the data we have obtained, we have integrated different lengths of peptides into 15mer based on the binding mode of peptide to MHC I. In order to demonstrate that our method is reliable to predict peptide-MHC binding, we compared our method with several popular methods. The experiments show the superiority of our method.

## Introduction

Many scholars try to find personalized treatment for melanoma and other cancers through major histocompatibility complex (MHC) (Kreiter et al., 2015; Bentzen et al., 2016; Johnson et al., 2016). Two successful phase I clinical trials proved that cancer vaccines are not a dream. These studies showed that 66.7 and 61.5% of resected melanoma patients have been cured during the period of 20–32 months and 12–23 months separately following vaccination (Ott et al., 2017; Sahin et al., 2017). These works were published in Nature, which have attracted more attention to personalized neoantigen vaccines (Chu et al., 2018).

Since neoantigens are ideal targets for immunotherapy, understanding the binding affinity between specific peptides and MHC alleles is an essential step in designing vaccines (Rolland et al., 2011; Cheng et al., 2017). The large number of peptide chains makes the research time-consuming and laborious. With the improvement of sequencing technology and bioinformatics, the binding affinity between predicted peptides and MHC alleles has become more flexible and economical (Jensen et al., 2018).

MHC is a gene family found in most vertebrate genomes and is closely related to the immune system. The MHC of humans is also known as human leukocyte antigen (HLA). There are two types of MHC; the first type of MHC processes internal decomposition of the protein (such as the virus), the second type of MHC is only located on antigen-presenting cells (APC), such as macrophages. For example, if there is bacterial invasion in the tissue, and the macrophage is swallowed, the bacterial fragments are prompted by MHC to the helper T cells to initiate an immune response. The regulated DNA is located on chromosome 6 (6p21.31) (Cheng et al., 2018; Cheng et al., 2019) and includes a series of tightly linked loci that are closely related to human immune system function (Neefjes et al., 2011). Some of these genes encode cell surface antigens, which are the “characteristics” that are not confusing for each person’s cells. They are the basis for the immune system to distinguish itself from foreign bodies. The HLA complex is located in the 21.31 region (6p21.31) on the short arm of chromosome 6, and is composed of 3.6 million base pairs. It is the region with the highest gene density and the most polymorphic region in human chromosomes. Known as “chemical fingerprints in humans”.

Recently, many researchers have focused on the field of predicting the binding affinity between peptide and MHC alleles. Some of them focused on the MHC-I and some of them focused on the MHC-II. There are also lots of tools and algorithms which are developed for this work. We classified these methods into three categories: Machine learning, neural network and deep learning.

Machine learning methods extracted features and constructed models to predict peptide-MHC interactions. Giguere S. et al. (2013) used kernel ridge regression to predict peptide-protein binding affinity. Uslan V and Seker H. (Uslan and Seker, 2016) used support vector regression (SVR) based on fuzzy model to do this work. Pavel P. Kuksa et al. (Kuksa et al., 2015) proposed a high-order semi-RBM to pretrain feed-forward high-order neural network (HONN). After that, high-order nuclear SVM was used to predict peptide-MHC binding. Although these methods can capture nonlinear interactions between different peptides, they fail to model the direct strong high-order interactions between features.

Recently, neural network (Hao et al., 2016; Hao et al., 2017) and deep learning (Peng et al., 2019a; Peng et al., 2019b) are the most common used methods in this field. Kasper W. Jorgensen (Jørgensen et al., 2014) developed a novel tool-NetMHCstab to predict stability of peptide-MHC complexes. They used Artificial neural network (ANN) to identify the stability of 10 different HLA class I molecules. Recently more studies tried to integrate peptides of different lengths into a machine-learning frame. These methods such as MHCflurry (O'Donnell et al., 2018) and NetMHCpan (Trolle et al., 2015) can involve more training data into their model and become popular tools for this task (Jurtz et al., 2017). NetMHC trained models for each MHC allele and this model is based on allele-specific approach (Andreatta and Nielsen, 2015). Whereas NetMHCIIpan (Jensen et al., 2018) is based on the pan-allele approach. Actually, they both used basic ANN with the immune epitope database (IEDB) (Vita et al., 2018; Salimi et al., 2019). NNAlign (Alvarez et al., 2018) which is a method based on neural network has been a common method to build models. Barra et al. (2018), Garde et al. ( 2019) all developed their own methods based on NNAlign. With the development of Mass Spectrometry (MS), the precision of identifying MHC ligands has been improved. Some researchers have proved that using MS data to do the training the model could be more robust. In the most recently released NetMHCpan 4.0 (Jurtz et al., 2017), they added MS data into their training set and improved their prediction accuracy.

Deep learning methods have shown their powerful ability of prediction and classification in recent years and have attracted more and more scholars’ attention (Peng et al., 2019c). Zeng and Gifford (2019) purposed a deep residual network-based computational approach that quantifies uncertainty in peptide-MHC affinity prediction. Sidhom et al. (2018) present Allele-Integrated MHC (AI-MHC), a deep learning architecture for human Class I and Class II MHC binding prediction. More researchers’ work (Bulik-Sullivan et al., 2019; Phloyphisut et al., 2019; Tran et al., 2019) have proved that deep learning methods have better performance than shallow neural networks.

The other important step to predict peptide-MHC binding affinity is extraction of feature. In the previous studies, most of the studies focused on the 9-mer peptides because most presented MHC class-I ligands are 9 mer (Bassani-Sternberg et al., 2015). However, for some alleles, they prefer other lengths of peptides. For example, Mamu-A2*05 preferentially binds 8-mer peptides (de Groot et al., 2017) and HLA-B*44:03 (Rist et al., 2013) prefers 10 and 11 mer peptides. Recently more and more researchers found methods to make all peptides into the same length so they can train their models with more data. Massimo Andreatta and Nielsen et al. (2015) added or deleted the primary sequence to ensure all the peptides are 9 mer. As a result, they involved the length of the deletion/insertion and the length and the composition of the peptide flanking regions in the feature. Youngmahn Han and Dongsup Kim (Han and Kim, 2017) considered each peptide as an image and each data in the feature is a pixel.

Although most previous studies have achieved high accuracy of prediction, there should be a novel method to use chemical properties of peptides to predict the binding affinity. In this paper, we used sequence comparison based on BLOSUM62 coding and to chemical properties of peptides extract feature and used convolutional neural network (CNN) to build models.

## Methods

### Feature Extraction

**Figure 1:**
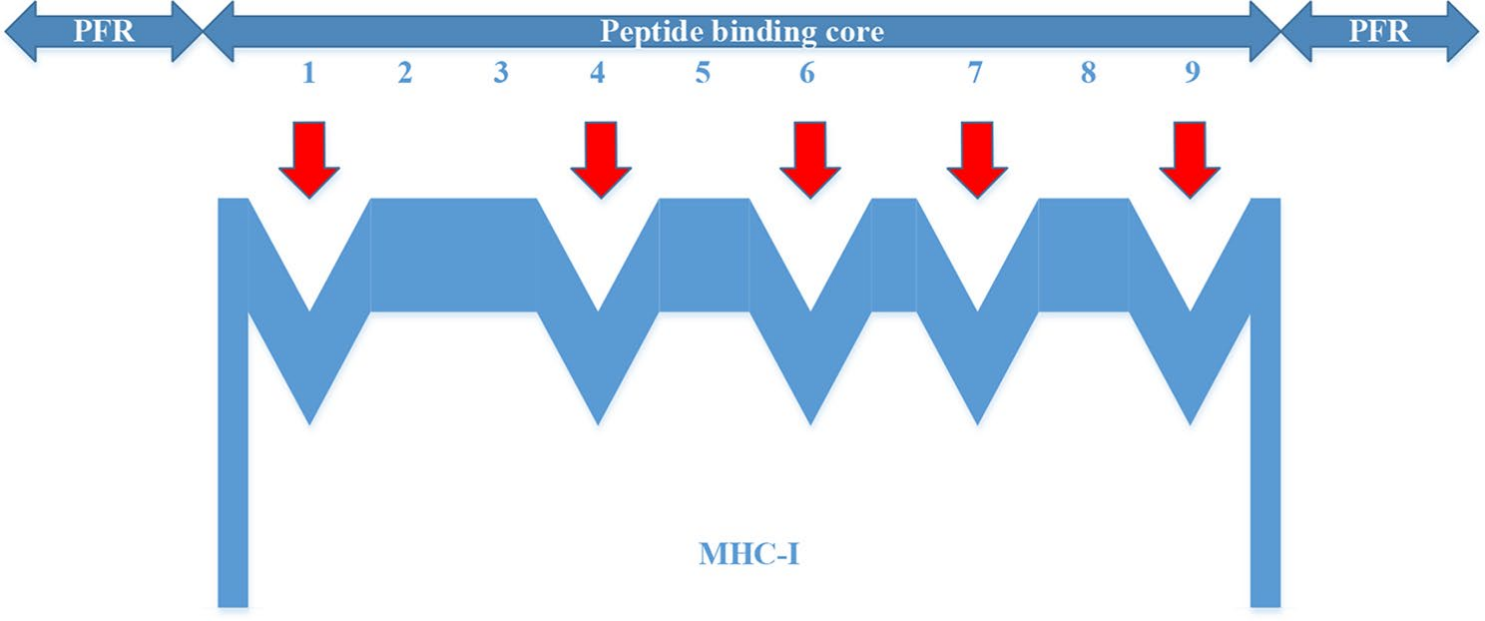
Binding of major histocompatibility complex -I molecules to affinity peptides.

**Figure 2:**
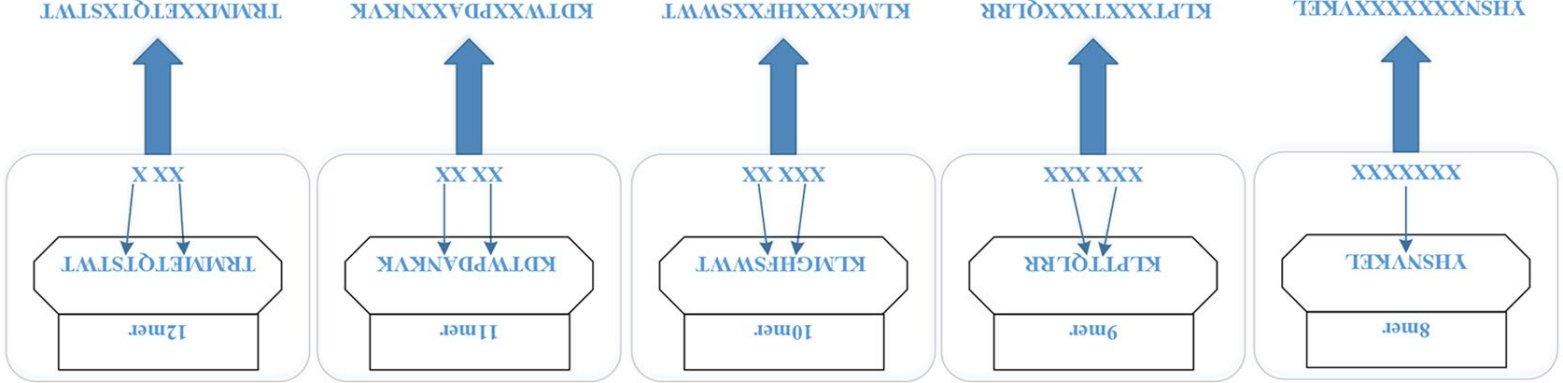
Encoding peptides of different lengths.

For the MHC-I complex, the alpha chain has three domains, wherein the grooves formed by the *α*1 and *α*2 regions can bind to an antigen peptide and the *α*3 region is a CD8 binding region. The *β* chain has only one domain of *β*2, forming a microglobulin structure. As shown in **Figure 1**, the binding core of nine amino acids plays a major role in the binding of the MHC-I molecule to the affinity peptide. At the same time, the peptide flanking residues (PFR) on both sides also plays a certain role in the binding. In the binding core, positions one, four, six, seven, nine are called “anchors” and play a more important role in binding than other locations. Based on this theory, we proposed a novel method that can convert the 8–14mer peptide to 15mer. Since one, four, six, nine are much more important than the other locations, we try to ensure that the two sequences of one to four and six to nine are not inserted into the new ‘amino acid’ (X). As we can see in **Figure 2**, we take 9–12mer peptide as an example. X is an artificial amino acid which is only related to itself and not related to the other 20 amino acids.

After converting all peptides to 15mer, all the peptides should be encoded by BLOSUM62 matrix (Styczynski et al., 2008). X is encoded as a vector of zeros but the score between X and itself is one. Then the feature of each peptide is a matrix 15*21.

The chemical properties of peptides have been reported to strongly affect the binding affinity. When the body is infected, inflammatory factors such as IFN-γ can change the β subunit composition of the proteasome 20S, making the proteasome more likely to cleave hydrophobic and alkalinous amino acids (so that the peptide is more easily bound to MHC-I). As said by Udaka et al. (1995) there is a general preference for hydrophobic amino acids. They also divided MHC-I into eight positions and found that the dominance of amino acids with hydrophobic side chains is unequivocal for some positions. Conversely, neutral or positively charged hydrophilic side chains are preferred in some other positions. In addition, Some positions allow hydrophobic as well as hydrophilic amino acids and appear to be less constrained than other positions.

Therefore, we proposed a novel way to extract the feature of peptides. We extracted four kinds of features: Sequence, Hydropathy index, Polarity, Length.

For the first feature: Sequence, we sorted the 21 kinds of amino acids by the BLOSUM62. ‘A’’, ‘R’, ‘N’, ‘D’, ‘C’, ‘Q’, ‘Ev, ‘G’, ‘H’, ‘I’, ‘L’, ‘K’, ‘M’, ‘F’, ‘P’, ‘S’, ‘T’, ‘W’, ‘Y’, ‘V’, ‘X’ are represented by the numbers 1 to 21 respectively.

For the second feature: Hydropathy index, we used Eisenberg consensus scale (ECS) (Eisenberg, 1984) to value each amino acid’s hydropathy index. X’s hydropathy index is zero. **Table 1** shows the score of every amino acid.

**Table 1:** Hydropathy Index of 21 amino acids.

Amino Acids	Hydropathy Index	Amino acids	Hydropathy Index
R	−2.5	K	−1.5
D	−0.9	Q	−0.85
N	−0.78	E	−0.74
H	0.40	S	−0.18
T	−0.05	P	0.12
Y	0.26	C	0.29
G	0.48	A	0.62
M	0.64	W	0.81
L	1.1	V	1.1
F	1.2	I	1.4
X	0		

For the third feature: Polarity, we divided 21 amino acids into five classes. According to the polarity of R group or the trend of interaction with water at physiological pH (approaching pH 7.0), they can be divided into non-polarity, polarity without charge, positive charge (alkalinity) and negative charge (acidity) (Wolfenden et al., 2015). X’s class is zero. **Table 2** shows the classification of every amino acid.

**Table 2:** Five Classes of amino acids based on polarity.

Class	Label	Amino acids
NONE	0	X
Polarity without charge	1	A, G, I, L, F, P, V
Non-polarity	2	N, C, Q, S, T, W, Y, M
Negative charge (acidity)	3	D, E
Positive charge (alkalinity)	4	R, H, K

For the fourth feature: Length, we use the length of peptide as a feature.

**Figure 3:**
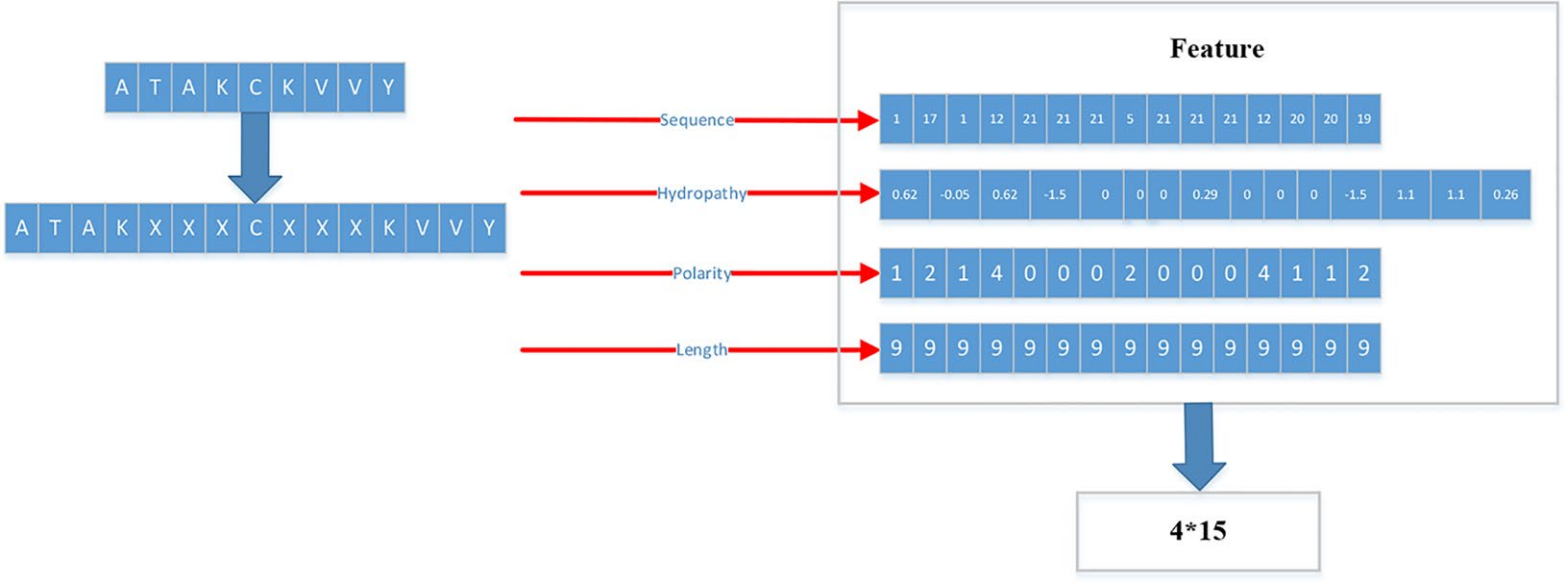
Detailed flow of generating training set and testing set.

The detailed flow is as in the following **Figure 3**.

As shown in **Figure 3**, each peptide would be encoded as a 4*15 matrix. N is the number of training set.

### Building Model by Convolutional Neural Network

Each peptide could be put into the CNN as a “picture” whose size is N*15. So we should set the structure of CNN firstly.

**Figure 4:**
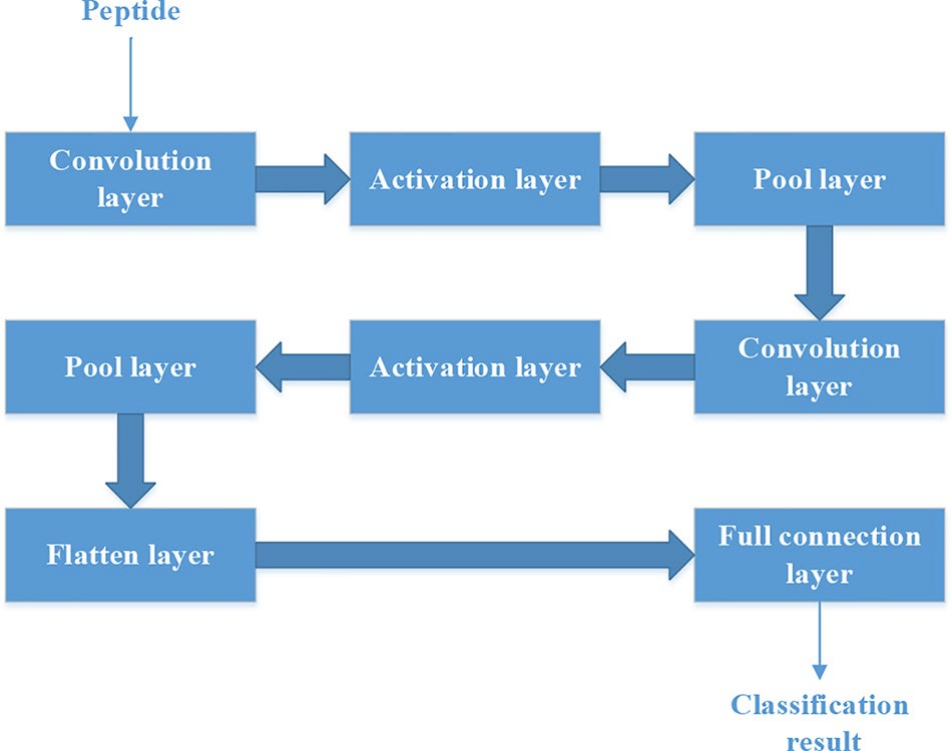
The structure of convolutional neural network.

**Figure 4** shows the structure of CNN. It contains two convolution layers. Each convolution layers have 20 filters. We used rectified linear unit (‘ReLu‘) as the activation function in the activation layer. ‘Max’ method is used in the Pool layer.

We built four models for different lengths of the peptides. We grouped the peptides by their length (L). The four groups are L < = 8, L = 9, L = 10 and L = > 11.

## Results

### Data Description

We downloaded three different datasets. The detailed information is shown in **Table 3**.

**Table 3:** Detailed information of data.

Name	Source
IEDB affinity data	Vita et al. (2018)
BD2013	Kim et al. (2014)
MS data	Abelin et al. (2017)

We totally obtained 525,672 peptides and the data include their allele, peptide, measurement value, measurement inequality, measurement type, measurement source, and original allele.

**Figure 5:**
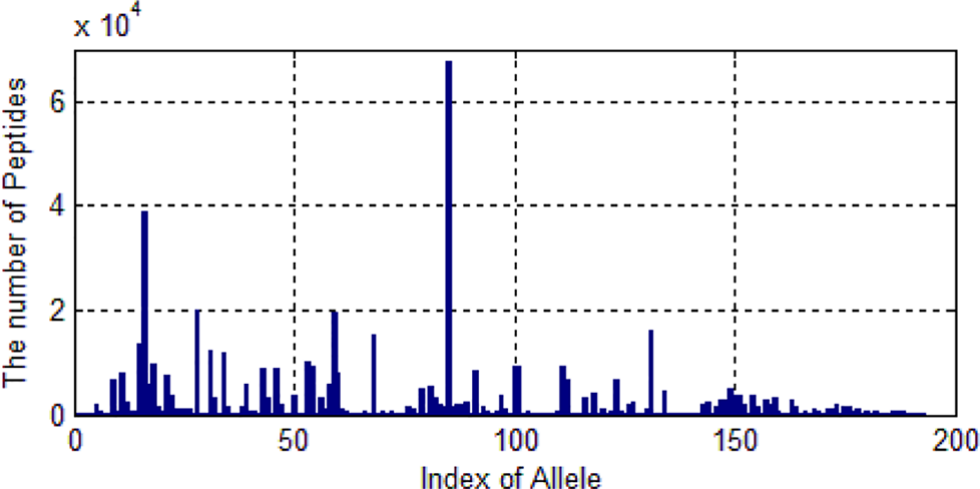
The distribution of the number of peptides of 193 alleles.

We only selected those alleles whose number of peptides are larger than 20. Then 522,268 peptides are left. These peptides belongs to 193 kinds of alleles. As shown in **Figure 5**, one allele has more than 60,000 peptide data and some alleles’ data are much smaller.

**Figure 6:**
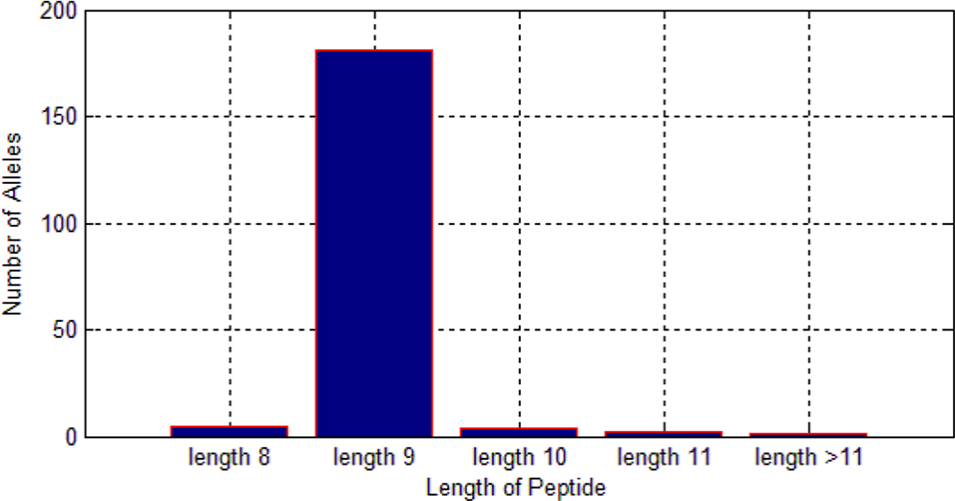
Length preference of 193 alleles.

Among these 522,268 peptides, there are 338,978 positive peptides. As we know, different alleles have different preferences for length of peptides. As shown in **Figure 6**, we found that most of the alleles prefer the length nine.

Therefore, it is much reasonable to put length of peptide into the feature matrix.

### Evaluation of the Convolutional Neural Network & Based on New Feature

We used both binding affinity (BA) data and eluted ligand (EL) data. After integrating the two data sets together, in order to prevent the uneven distribution of the negative and positive peptides, we sorted the data in disorder. Then, we did fivecross validation.

HLA type alleles are the data we care about most. There are 43 HLA-A alleles and 82 HLA-B alleles in our dataset. In the Youngmahn Han and Dongsup Kim’s paper (Han and Kim, 2017), they used Deep CNN to compare with NetMHCPan, SMM(47), ANN, and PickPocket (Zhang et al., 2009). We used their statistical data and evaluated our CNN which is based on the novel feature. We call our method CNN-NF.

F1 score is used to evaluate models. It can be calculated as:

(1) $$F1=\frac{2\mathrm{TP}}{2\mathrm{TP}+\mathrm{FN}+\mathrm{FP}}$$

Here, true positive (TP) denotes positive samples whose predictions are positive. false negative (FN) denotes positive samples whose predictions are negative. false positive (FP) denotes negative samples whose predictions are positive.

As we can see in **Table 4**, **Tables 4A, B** summarize the prediction results for HLA-A and HLA-B alleles, respectively. The mean values of the F1 Score of the CNN-NF were 0.643 and 0.692. The values are slightly higher than those of other methods. In addition to that, the standard deviation of the two experiments are lower than those of other methods’ either. It means that CNN-NF is more stable.

**Table 4:** Prediction results for human leukocyte antigen-1 (HLA-I) alleles(A).

(A) Summary of prediction results for HLA-A alleles (F1 Score)						
	CNN-NF	DCNN	NetMHCPan	SMM	ANN	PickPocket
Mean	0.643	0.638	0.608	0.601	0.579	0.561
Median	0.603	0.696	0.667	0.667	0.667	0.625
Standard Deviation	0.166	0.23	0.267	0.250	0.286	0.318
**(B) Summary of prediction results for HLA-B alleles (F1 Score)**						
	CNN-NF	DCNN	NetMHCPan	SMM	ANN	PickPocket
Mean	0.692	0.593	0.606	0.578	0.606	0.560
Median	0.621	0.667	0.625	0.615	0.643	0.593
Standard Deviation	0.228	0.286	0.286	0.302	0.290	0.277

**Figure 7:**
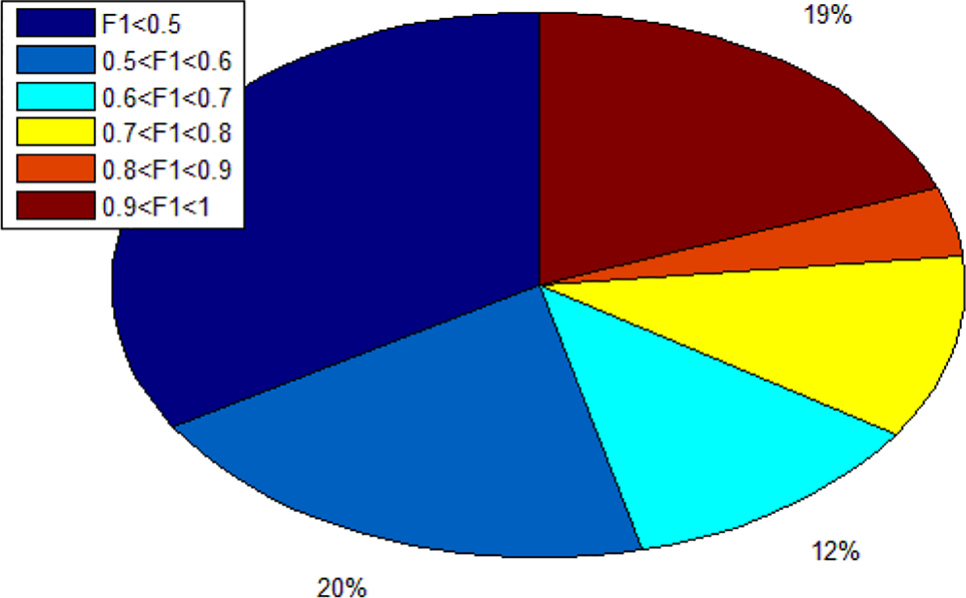
The distribution ratio of F1 score.

Since we totally obtain 193 alleles, we calculated 193 F1 scores. As shown in **Figure 7**, there are 19% alleles whose F1 score are more than 0.9. In addition, there are 34% alleles whose F1 score are lower than 0.5. We can know that different alleles have different accuracy and even polarization.

**Figure 8:**
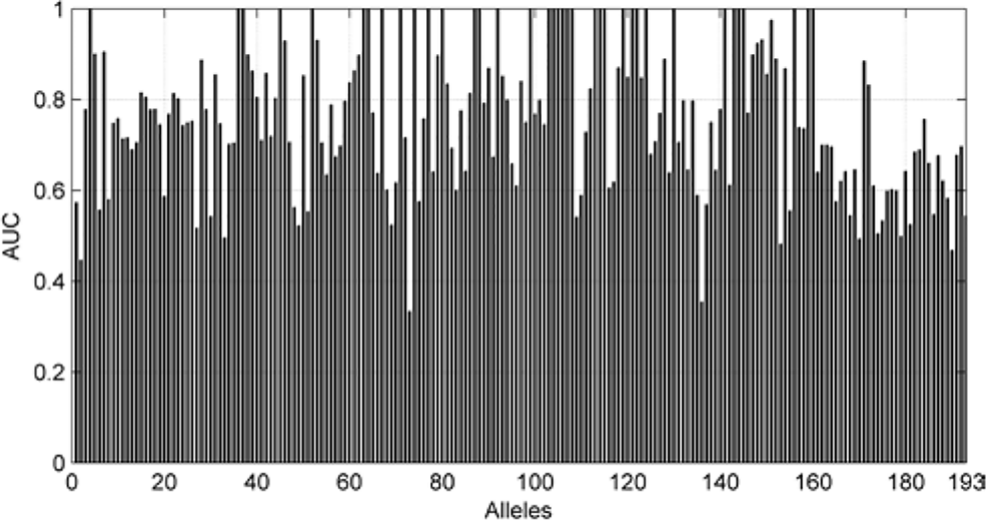
AUC of each allele.

We also are interested in the area under curve (AUC) of the 193 allele experiments. We draw **Figure 8** for each allele’s performance of AUC and another figure for the distribution of AUC in 193 experiments.

**Figure 9:**
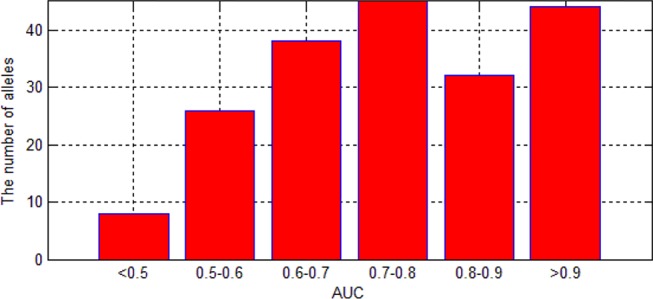
The distribution of AUC in 193 experiments.

As we can see in **Figure 9**, although there are some alleles whose accuracy are lower than 0.5, most of the alleles have an accuracy more than 0.7. The low accuracy of some alleles may be due to the small amount of data. It may also be caused by the extreme imbalance of data.

### Peptide-Length Preference Of Major Histocompatibility Complex Molecules

Although we have known that most of the alleles mostly prefer the nine length peptide, different alleles have different preferences in 8,9,10,11,12,13,14,15mer peptides. We should verify the ability of our method to capture peptide long preferences for different MHC molecules. Therefore, we randomly generated 10,000 peptides for each MHC molecules. These 10,000 peptides’ length range from 8 to 15. The number of peptides of each length is the same so each length has 1,250 peptides. Then we put these artificial peptides into the models and the models would tell us the probability of being positive. We selected the top 2% probabilities and calculated the distribution of different lengths.

**Figure 10:**
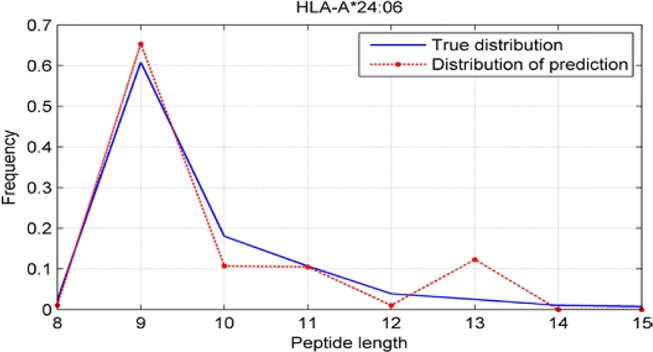
Predicted length preference of HLA-A*24:06.

**Figure 11:**
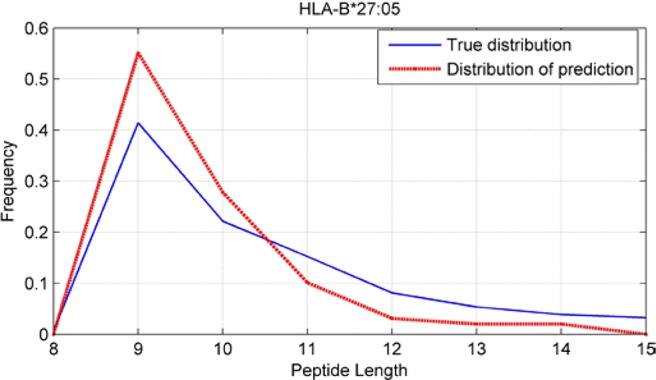
Predicted length preference of HLA-B*27:05.

**Figure 12:**
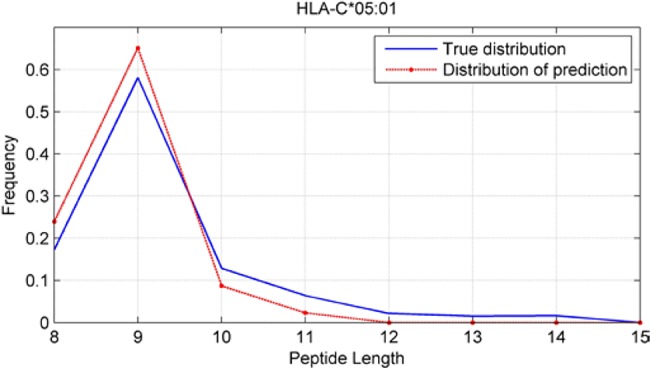
Predicted length preference of HLA-C*05:01.

As shown in **Figures 10**–**12**, we randomly selected an allele for each HLA-A, B, and C coding site to verify the ability of our method to capture peptide long preferences for different MHC molecules.

CNN-NF prefer to identify the 9mer peptide as the binding peptide. Besides, if the number of the specific length peptide is small, CNN-NF can hardly give a high score. We can consider this phenomenon as a way that CNN guarantee the training accuracy.

## Conclusions

In this paper, we purposed a novel method for peptide-MHC-I binding prediction. Since deep learning is developing fast, we consider that it has more advantages than shallow neural networks. The other more important reason to introduce CNN to this field is that the most commonly used format of feature for each peptide is a matrix. Therefore most researchers usually first convert the feature matrix into a line or a column. However, CNN could find out the real feature of each peptide by the initial feature matrix. In brief, CNN is more suitable for predicting peptide-MHC-I binding affinity.

Another novel thought of our paper is the way of extracting feature. The most common way to extract feature is based on BLOSUM nowadays. Although BLOSUM is a typical way to do sequence alignment, the order of the sequence and the characteristic of the acid amino would undoubtedly affect the binding of peptides to genes. Therefore, we extracted four kinds of feature for each peptide. They are the order of the sequence, hydropathy index, polarity, and length.

Our work flow can be concluded in three steps. Firstly, we convert every length of peptide into 15mer based on the binding mode of peptide to MHC I. Then, we extracted feature of each peptide based on the order of the sequence, hydropathy index, polarity, and length. For each peptide, the feature of it should be a matrix with 4 * 15 dimension. Finally, we built a frame of CNN and put these features and their corresponding label into it.

We put three data sets together and obtain 525,672 peptides. We built model for each alleles so we totally built 193 models. To verify the accuracy of our model, we did five cross validation. We compared our method with DCNN, NetMHCPan4.0, SMM, ANN and PickPocket. In most cases, the accuracy of CNN-NF is higher than that of other methods. In addition, we also use our model to test the preference of different alleles to length. The length preference obtained by prediction is very close to the true preference.

## Data Availability Statement

Publicly available datasets were analyzed in this study. This data can be found here: http://tools.iedb.org/mhci/download/. Code and data are available at https://github.com/zty2009/MHC-I/tree/master.

## Author Contributions

TZh wrote this paper and did experiments. LC provided important ideas. This whole work is guided by TZa and YH. TZa and YH also provided all the materials and environment to complete this work.

## Funding

TZ and YH are the corresponding authors. TZh and LC are co-first authors. This work was supported by the National Natural Science Foundation of China (Nos: 61571152 and 61502125), the National High-tech R&D Program of China (863 Program) [Nos: 2014AA021505, 2015AA020101, 2015AA020108], the National Science and Technology Major Project [Nos: 2013ZX03005012 and 2016YFC1202302], Heilongjiang Postdoctoral Fund (Grant No. LBH-Z15179), and China Postdoctoral Science Foundation (Grant No. 2016M590291).

## Conflict of Interest

The authors declare that the research was conducted in the absence of any commercial or financial relationships that could be construed as a potential conflict of interest.
